# FLAIR vascular hyperintensities in acute ischemic stroke: from collateral-related imaging findings to a candidate dynamic hemodynamic imaging phenotype

**DOI:** 10.3389/fneur.2026.1960930

**Published:** 2026-10-14

**Authors:** Mingqian Qin, Lei Wang, Guosheng Han

**Affiliations:** Affiliated Hospital of Shandong Second Medical University, Weifang, China

**Keywords:** acute ischemic stroke, collateral circulation, FLAIR vascular hyperintensities, FVHs, FVHs-DWI mismatch, hemodynamic adaptation, large vessel occlusion (LVO), reperfusion therapy

## Abstract

With advances in reperfusion therapies for acute large vessel occlusion (LVO), stroke imaging evaluation has progressively shifted from time-based treatment selection toward comprehensive assessment of tissue status and hemodynamic adaptation. However, current imaging approaches primarily characterize vascular patency, perfusion status, and tissue injury, while remaining limited in their ability to directly capture the dynamic regulatory processes of cerebral circulation after arterial occlusion. In patients with LVO, ischemic tissue outcome is determined not only by the occlusion site and time to reperfusion but also by collateral circulation capacity, cerebral hemodynamic status, and tissue tolerance to ischemia. Therefore, imaging biomarkers capable of reflecting cerebral circulatory adaptation under ischemic conditions are of substantial research interest. FLAIR vascular hyperintensities (FVHs) are MRI findings characterized by linear or serpentine hyperintense signals along the cortical surface, within cortical sulci, or along the Sylvian fissure on fluid-attenuated inversion recovery (FLAIR) sequences. They are generally associated with reduced distal blood flow velocity, prolonged blood transit time, and hemodynamic alterations mediated by leptomeningeal collateral pathways. In recent years, numerous studies have investigated the role of FVHs in the diagnosis of acute ischemic stroke, evaluation of reperfusion therapy, and prediction of clinical outcomes. However, the clinical significance of FVHs remains incompletely understood. Some studies have suggested that FVHs are associated with favorable collateral status and smaller infarct volumes, whereas others have demonstrated that FVHs may also occur in patients with more pronounced hemodynamic impairment. These discrepancies suggest that FVHs may not simply represent favorable collateral circulation or ischemic severity, but may instead reflect the dynamic hemodynamic response of the cerebral circulation to reduced perfusion following arterial occlusion. From the perspective of precision stroke imaging, this review systematically summarizes the mechanisms underlying FVH formation, their biological significance, and clinical implications. Particular emphasis is placed on the relationships between FVHs and collateral circulation, FVHs-DWI mismatch, ischemic tissue status, and potential benefit from reperfusion therapy. Furthermore, current challenges related to FVH standardization and clinical translation are discussed, with particular emphasis on their potential role as a candidate dynamic hemodynamic imaging phenotype reflecting physiological hemodynamic adaptation.

## 1 Introduction

Treatment strategies for acute ischemic stroke have progressively shifted from time-window-based approaches toward precision decision-making guided by tissue status and hemodynamic characteristics, driven by advances in endovascular therapy. In patients with acute LVO, mechanical thrombectomy significantly improves functional outcomes; however, the magnitude of benefit remains influenced by infarct core volume, residual perfusion status, and collateral circulation capacity (1–5). The DAWN and DEFUSE 3 trials demonstrated that patients selected according to imaging criteria, including a small infarct core or clinical–imaging mismatch, could achieve substantial benefit from reperfusion therapy beyond conventional treatment windows (1, 2). Nevertheless, current imaging modalities primarily characterize vascular anatomy, tissue perfusion, and injury extent, while their ability to directly capture the dynamic adaptive responses of the cerebral circulation after acute arterial occlusion remains limited (4, 5). Therefore, imaging markers capable of capturing distal flow redistribution and hemodynamic adaptation after occlusion may provide complementary information beyond existing vascular and perfusion assessments.

FVHs are common vascular-related imaging findings observed on MRI in patients with acute ischemic stroke. They typically appear as linear or serpentine hyperintense signals along the cortical surface, within cortical sulci, or along the Sylvian fissure distal to an occluded artery. Previous studies have suggested that FVHs are associated with reduced blood flow velocity, prolonged blood transit time, and flow-related intravascular signal alterations, indicating their potential to reflect distal hemodynamic abnormalities during acute ischemia (6–11). Because FVHs frequently occur in patients with LVO and their spatial distribution partially overlaps with territories supplied by leptomeningeal collateral pathways, early studies primarily interpreted FVHs as collateral-related imaging findings (12).

Subsequently, Lee et al. (12) reported that distal FVHs were associated with larger perfusion–diffusion mismatch and smaller ischemic lesion volumes, suggesting that FVHs may reflect residual flow mediated by leptomeningeal collaterals distal to the occlusion and may be associated with preserved potential tissue recovery. In the era of endovascular therapy, increasing attention has been directed toward the relationship between FVHs, collateral status, tissue recovery after reperfusion, and functional outcomes. These studies have suggested that FVHs may provide complementary imaging information associated with tissue recovery and clinical outcomes after reperfusion therapy (13–16). However, as research on FVHs has expanded, their relationships with collateral status, perfusion characteristics, and clinical outcomes have shown inconsistent patterns, suggesting that the biological significance of FVHs extends beyond conventional collateral assessment.

Current evidence indicates that the biological significance of FVHs cannot be fully explained by collateral circulation status alone. Some studies have demonstrated that FVHs are associated with favorable collateral circulation and relatively preserved tissue conditions, whereas others have shown that FVHs can also occur in patients with more severe hemodynamic impairment. These findings indicate that FVHs do not have a simple one-to-one relationship with collateral status or actual perfusion levels (12, 13, 17, 18). Importantly, FVHs should not be interpreted as a uniform marker of collateral function, because their clinical meaning depends on imaging timing, spatial relationship with ischemic tissue, and reperfusion status. In the pretreatment setting, FVHs located outside the DWI-defined infarct core may reflect collateral-supported residual perfusion with delayed distal blood transit, suggesting the presence of hemodynamically supported but vulnerable tissue (12, 14, 19, 20). In contrast, persistent FVHs after endovascular reperfusion may represent incomplete restoration of distal flow, residual hemodynamic impairment, or post-reperfusion vascular alterations despite successful recanalization (15, 16). Therefore, FVHs represent a context-dependent imaging phenotype reflecting distal hemodynamic adaptation rather than a direct surrogate marker of collateral status or tissue viability (17, 18, 21, 22).

In this context, the term “dynamic” refers to the physiological adaptation of cerebral circulation after arterial occlusion rather than temporal changes captured by serial imaging. FVHs reflect the interaction among collateral recruitment, redistribution of residual blood flow, and distal hemodynamic alterations, which collectively determine intravascular signal expression on FLAIR imaging. Therefore, FVHs should not be interpreted as a direct measure of collateral anatomy or tissue perfusion. Instead, the term “imaging phenotype” emphasizes that FVHs represent an observable imaging manifestation resulting from the interaction between vascular status, blood flow conditions, and tissue response during acute ischemia. Compared with conventional imaging markers that primarily assess collateral vessels or perfusion parameters, FVHs provide complementary information regarding distal hemodynamic adaptation after arterial occlusion.

Based on these observations, FVHs may be better understood as an imaging phenotype reflecting dynamic cerebral circulatory adaptation after arterial occlusion. However, this interpretation requires further validation through direct assessment of blood flow, perfusion parameters, and multimodal imaging approaches.

The complex associations among FVHs, collateral status, perfusion characteristics, and clinical outcomes suggest that FVHs represent an imaging phenotype reflecting the interaction among vascular status, blood flow, and tissue response after acute arterial occlusion. From the perspective of precision stroke imaging, this review summarizes the mechanisms underlying FVH formation, their biological significance, and their clinical applications. Particular emphasis is placed on the relationships between FVHs and collateral circulation, FVHs-DWI mismatch, ischemic tissue status, and potential benefit from reperfusion therapy. Furthermore, we discuss future directions for FVHs research, focusing on their potential role as a candidate dynamic hemodynamic imaging phenotype. Future studies involving imaging standardization, multimodal imaging integration, and prospective validation are required to clarify the clinical value of FVHs within comprehensive imaging frameworks for acute ischemic stroke.

A recent comprehensive review by Ouyang et al. (23). summarized the methods, imaging characteristics, underlying mechanisms, and clinical significance of hyperintense vascular signs on MRI across cerebrovascular diseases. This work provided an important overview of vascular-related hyperintense signals and their potential applications. However, the interpretation of FVHs in acute ischemic stroke remains particularly challenging because their biological meaning varies according to imaging timing, relationship with ischemic tissue, and reperfusion status. In contrast to a general description of hyperintense vascular signs, the present review specifically focuses on acute LVO stroke and proposes that FVHs should be interpreted as a context-dependent dynamic hemodynamic imaging phenotype rather than a direct surrogate marker of collateral status or tissue viability.

## 2 Biological mechanisms underlying FVH formation

The ischemic process following acute LVO is not simply characterized by complete loss of distal blood flow; rather, it represents a complex circulatory adaptation process influenced by reduced perfusion pressure, redistribution of blood flow, and recruitment of collateral pathways. Following occlusion of a major cerebral artery, reduced perfusion pressure within the distal vascular bed promotes recruitment of pre-existing leptomeningeal collateral pathways, which provide alternative routes of blood supply to maintain residual perfusion within ischemic regions. Because the anatomical architecture, recruitment capacity, and efficiency of collateral circulation vary substantially among individuals, occlusion at the same vascular location may result in different degrees of hypoperfusion and tissue injury (4, 5). FVHs are thought to arise during this dynamic circulatory adaptation process, reflecting the complex interaction among collateral recruitment, maintenance of residual blood flow, and distal hemodynamic impairment (4, 5, 12).

FVHs appear as linear or serpentine hyperintense signals distributed along the cortical surface, within cortical sulci, and along the Sylvian fissure on FLAIR images. Their development occurs in the setting of reduced perfusion pressure after arterial occlusion, subsequent collateral recruitment, and altered distal hemodynamic conditions (10) (Figure 1). These findings are typically located distal to acute arterial occlusion and are associated with reduced blood flow velocity and prolonged blood transit time within leptomeningeal collateral pathways (9–11, 24). Because FVHs primarily reflect alterations in blood motion and intravascular signal characteristics rather than structural vascular abnormalities, their biological significance extends beyond conventional vascular morphological assessment.

**Figure 1:**
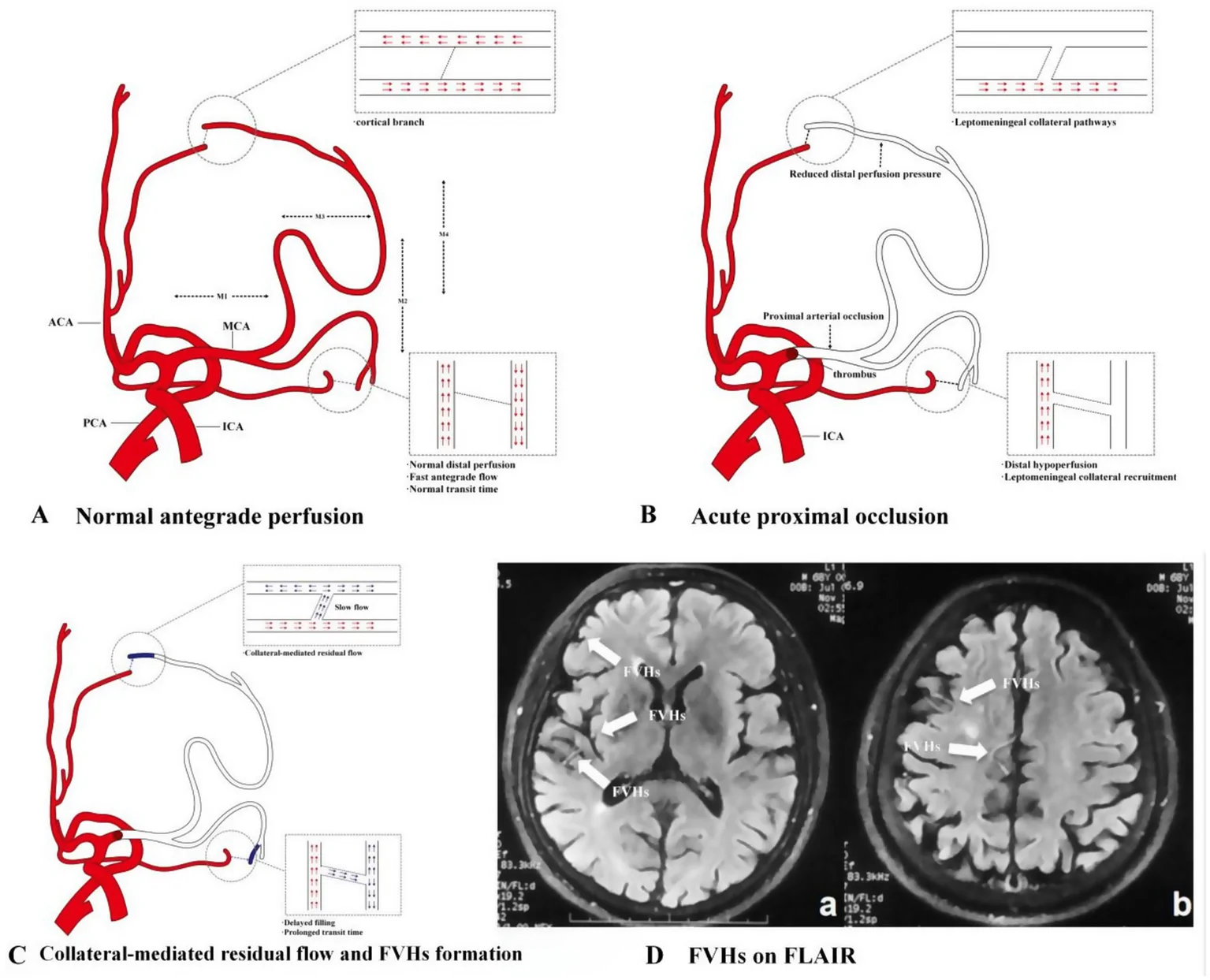
Dynamic hemodynamic basis and MRI manifestation of FLAIR vascular hyperintensities in acute ischemic stroke. **(A)** Under physiological conditions, the middle cerebral artery (MCA) supplies the distal vascular bed through antegrade blood flow with preserved perfusion pressure and normal transit time. **(B)** Acute proximal arterial occlusion decreases distal perfusion pressure and induces recruitment of leptomeningeal collateral pathways. **(C)** Collateral-mediated residual flow maintains partial distal perfusion but is characterized by reduced flow velocity, delayed filling, and prolonged transit time within distal vessels. These hemodynamic alterations are considered to contribute to the development of FLAIR vascular hyperintensities (FVHs). **(D)** Representative axial FLAIR images demonstrate tubular or serpentine hyperintense vessels along the Sylvian fissure and cortical sulci, consistent with FVHs. FVHs are considered to reflect abnormal intravascular signals associated with slow-flow conditions in distal vessels during collateral-mediated hemodynamic adaptation.

FVHs were initially considered to reflect reduced blood flow within distal vessels in ischemic territories. Noguchi et al. (25) described hyperintense vascular signals on FLAIR imaging in patients with cerebral infarction and suggested that these findings were associated with abnormal post-ischemic flow states (24). Subsequently, Cosnard et al. (6) demonstrated that abnormal vascular hyperintensity on FLAIR imaging in patients with ischemic stroke may reflect alterations in intravascular flow conditions. Toyoda et al. (7) further proposed that FLAIR intra-arterial hyperintense signals in acute ischemia were associated with reduced blood flow velocity and prolonged blood residence time, suggesting that slow flow and delayed filling of distal vessels contribute to FVH formation (26).

From the perspective of MRI signal generation, reduced blood flow velocity or prolonged intravascular blood residence decreases flow-related signal attenuation, resulting in persistent intravascular signal and hyperintense appearance on FLAIR images. Therefore, FVHs are generally considered to reflect changes in blood motion and intravascular signal behavior rather than intrinsic structural alterations of vessels (6, 7, 10). This characteristic provides FVHs with hemodynamic-related imaging properties. Angiographic studies further demonstrated that FVHs are associated with slow-flow conditions rather than intravascular thrombus, supporting the interpretation of FVHs as a hemodynamic imaging phenomenon (27).

However, slow flow alone does not fully explain FVH formation. Following acute arterial occlusion, recruitment of leptomeningeal collateral pathways provides alternative blood supply to ischemic regions while simultaneously altering distal hemodynamic conditions (4, 5, 12). Although collateral circulation is based on pre-existing anatomical connections, its actual contribution to tissue perfusion depends on pressure gradients induced by arterial occlusion, the degree of collateral recruitment, and hemodynamic efficiency (4, 5). After collateral recruitment, blood can bypass the occluded segment and enter the distal vascular bed; however, because collateral pathways are longer and associated with increased vascular resistance, blood reaching the distal circulation may exhibit reduced velocity and delayed arrival time (18). This state, characterized by preserved residual perfusion despite prolonged blood transit through the distal circulation, may contribute to FVH formation (4, 5, 26).

The relationship between FVHs and collateral circulation further highlights the complex biological characteristics of this imaging finding. Lee et al. (12) demonstrated that distal FVHs were associated with collateral circulation status and perfusion–diffusion mismatch, suggesting that FVHs may reflect residual flow conditions after arterial occlusion. Nave et al. (13) further reported associations among FVHs, collateral status, and functional outcomes after endovascular therapy, supporting the potential clinical relevance of FVHs. However, these studies also indicate that FVHs cannot simply be considered a surrogate marker of favorable collateral circulation. Mahdjoub et al. (17) showed that FVHs and collateral status were not completely concordant, as FVH appearance was influenced by occlusion location, collateral function, and the overall hemodynamic environment.

Taken together, the formation of FVHs may represent an imaging manifestation of adaptive regulation within the cerebral circulation in response to hypoperfusion after acute arterial occlusion. Arterial occlusion reduces perfusion pressure, triggering collateral recruitment and redistribution of blood flow; meanwhile, reduced distal flow velocity and delayed filling enhance intravascular signal visibility on FLAIR imaging. Therefore, FVHs may simultaneously reflect preserved residual blood flow and distal hemodynamic limitation. This conceptual framework provides a theoretical basis for further investigating the relationship between FVHs, tissue salvageability, treatment response, and future dynamic hemodynamic imaging assessment.

## 3 FVHs and collateral circulation

Collateral circulation is a critical determinant of distal perfusion maintenance and tissue injury progression after acute LVO. Following occlusion of a major cerebral artery, leptomeningeal anastomoses can redirect blood flow from adjacent vascular territories into hypoperfused regions, thereby delaying infarct core expansion and partially preserving potential tissue recovery within ischemic regions. However, the contribution of collateral circulation to distal perfusion depends not only on the presence of vascular connections but also on multiple factors, including occlusion location, perfusion pressure gradients, vascular reactivity, and individual metabolic status (4, 5, 28). Therefore, the anatomical extent of collateral vessels visualized on vascular imaging does not necessarily correspond to their functional perfusion efficiency, which may partly explain discrepancies among different collateral assessment methods.

Digital subtraction angiography (DSA) can demonstrate the extent and temporal characteristics of collateral filling; however, its invasive nature limits routine clinical use. Computed tomography angiography (CTA) enables rapid assessment of the spatial distribution of leptomeningeal collaterals but may be influenced by acquisition timing and scoring methodology (5). Perfusion imaging provides a tissue-level assessment of hypoperfusion extent and severity. Among perfusion parameters, the hypoperfusion intensity ratio (HIR) reflects the proportion of severely hypoperfused tissue within the overall hypoperfused region and has been associated with infarct progression and functional outcomes (29). These imaging approaches assess different aspects, including vascular anatomy, flow timing, and tissue perfusion, and should not be considered interchangeable measures of collateral status.

Unlike CTA or DSA, which primarily evaluate the anatomical availability and filling characteristics of collateral vessels, FVH assessment mainly reflects abnormal vascular signal distribution within the distal vascular bed beyond an occlusion (Figure 2). Therefore, FVHs may represent the hemodynamic state of the distal vascular bed after collateral recruitment rather than simply reflect the quantity of collateral vessels. In addition to FVH presence and extent, the topographic distribution of FVHs within the MCA territory may provide additional information regarding distal vascular involvement and revascularization-related hemodynamic changes (30).

**Figure 2:**
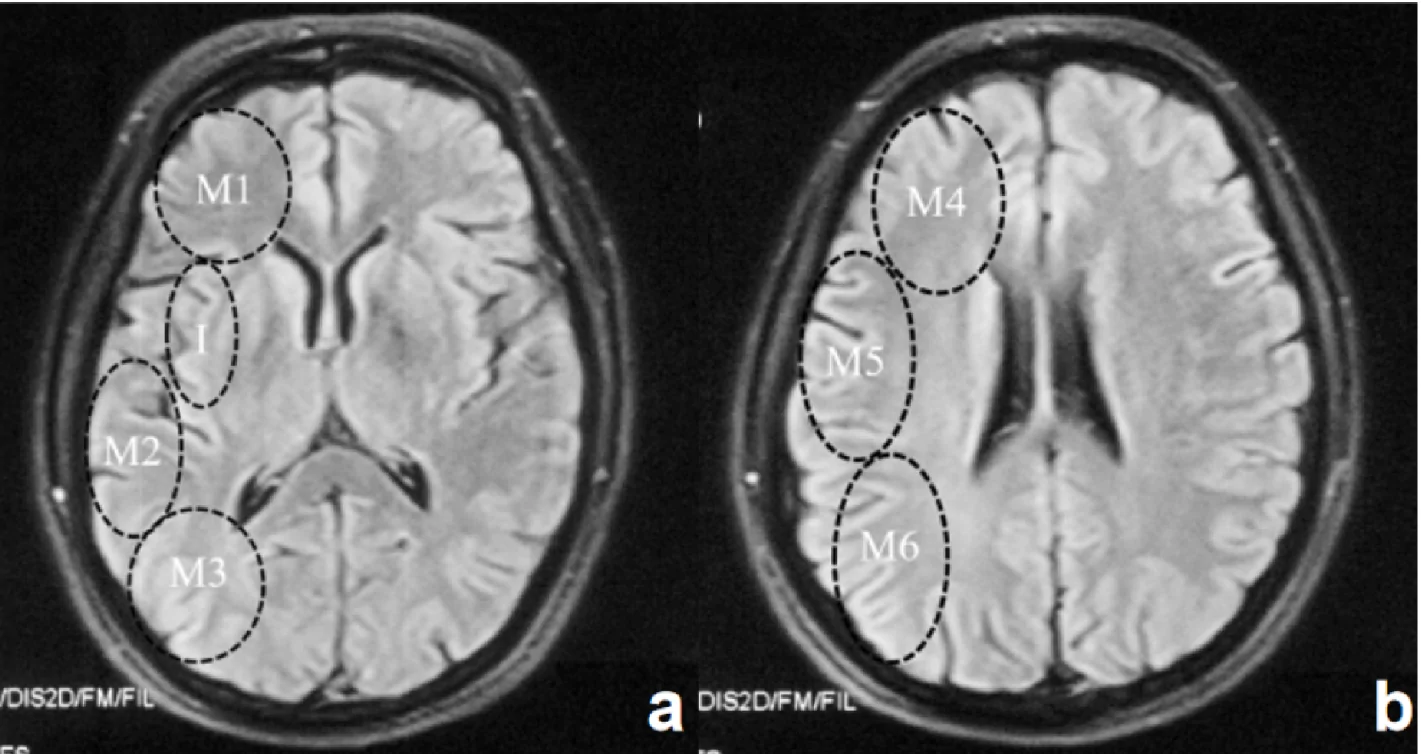
Regional assessment of FVHs. FVHs were assessed on two axial imaging levels encompassing seven anatomical regions. Level 1: The basal ganglia level, including the insular region (I), anterior MCA cortical territory (M1), lateral insular cortex (M2), and posterior MCA cortical territory (M3). Level 2: The lateral ventricular level, including the cortex adjacent to the anterior MCA territory (M4), cortex adjacent to the lateral insular cortex (M5), and cortex adjacent to the posterior MCA territory (M6). This regional division enables semi-quantitative evaluation of FVH distribution across the MCA territory.

The association between FVHs and collateral circulation is initially suggested by their spatial distribution distal to arterial occlusion. Lee et al. (12) reported that, in patients with proximal middle cerebral artery occlusion, more extensive distal FVHs were associated with larger perfusion–diffusion mismatch volumes and smaller ischemic lesion volumes. The authors proposed that hyperintense vessels distal to the occlusion may represent slow-flow conditions resulting from collateral-mediated blood supply through leptomeningeal pathways (12). FVHs were therefore considered a collateral-related imaging finding that allows indirect visualization of distal flow alterations without the need for contrast administration. However, this study did not establish an equivalent relationship between FVHs and angiographic collateral grading (12).

Subsequent studies further supported the association between FVHs and collateral-related imaging characteristics from both radiological and clinical perspectives. Mahdjoub et al. (17) used the HIR as an indicator of hemodynamic impairment and analyzed 244 patients with middle cerebral artery stroke who underwent MRI before reperfusion therapy. They found that more extensive FVHs were associated with a higher likelihood of lower HIR values, and this association remained after adjustment for potential confounding factors. Nave et al. (13) analyzed 116 patients with M1 segment occlusion undergoing endovascular therapy and demonstrated that FVH-ASPECTS scores were associated with angiographic collateral status and 3-month functional outcomes, suggesting that FVH burden may contain information related to tissue recovery after reperfusion. However, differences in scoring direction, anatomical segmentation, and collateral reference standards among studies prevent the establishment of a universal FVH-based collateral grading threshold.

The statistical association between FVHs and favorable collateral status does not indicate that these concepts are interchangeable. FVH expression depends on reduced blood flow velocity, prolonged blood transit time, and flow-related signal alterations (6, 7); Similar FVH patterns may represent relatively preserved yet delayed collateral flow or may occur in vascular territories with more severe perfusion impairment. Furthermore, occlusion location, imaging timing, recanalization status, and scoring methodology may influence the observed extent of FVHs. Therefore, FVHs are better considered a hemodynamic imaging manifestation associated with collateral circulation rather than an independent and stable collateral grading tool (18).

The information provided by FVHs does not represent the anatomical number of collateral vessels, but rather the presence of abnormal vascular signals detectable by FLAIR imaging within the distal vascular bed after occlusion. CTA and DSA primarily characterize collateral pathway availability, filling extent, and temporal flow patterns, whereas FVHs may provide complementary information regarding slow flow and residual perfusion after collateral recruitment (5, 8, 9, 12, 18). This framework also provides a theoretical basis for further exploring the relationship between FVHs and salvageable ischemic tissue. Importantly, previous studies have reported heterogeneous associations between FVHs, collateral status, tissue conditions, and clinical outcomes. These discrepancies may partly result from differences in imaging timing, occlusion location, reperfusion status, and reference standards used for evaluating collateral circulation or tissue injury (Table 1).

**Table 1:** Representative studies investigating the biological mechanisms and clinical significance of FLAIR vascular hyperintensities in acute ischemic stroke.

Study	Main research question	Key findings	Current interpretation
Cosnard et al. (6)	Initial characterization of FVHs	FLAIR vascular hyperintensities were associated with abnormal vascular signals during acute ischemia	Established FVHs as flow-related vascular abnormalities
Toyoda et al. (7)	Mechanism of FVH formation	Reduced flow velocity and prolonged intravascular residence time contributed to FVH appearance	Supported the slow-flow mechanism underlying FVH formation
Sanossian et al. (27)	Whether FVHs represent thrombus or slow flow	Angiography demonstrated that FVHs were related to slow flow rather than thrombus	Reinforced FVHs as a hemodynamic rather than structural vascular marker
Lee et al. (12)	Relationship between FVHs and collateral circulation	Distal FVHs were associated with perfusion–diffusion mismatch and collateral-related residual flow	FVHs may reflect collateral-mediated residual flow but not collateral anatomy itself
Haussen et al. (19)	FVHs beyond DWI lesion and tissue status	FVHs beyond DWI abnormalities were associated with perfusion–diffusion mismatch	FVHs may indicate residual hemodynamic support but do not directly define tissue viability
Legrand et al. (32)	Whether FVHs beyond the DWI lesion represent ischemic penumbra	FVHs extending beyond DWI lesions were investigated as a marker of potentially salvageable tissue	FVHs-DWI mismatch reflects hemodynamic alteration rather than a direct surrogate of ischemic penumbra
Mahdjoub et al. (17)	Whether FVHs represent good collaterals	FVHs were not completely concordant with angiographic collateral status	Challenged the concept of FVHs as a direct collateral marker
Nave et al. (13)	FVHs and outcome after EVT	FVH burden was associated with collateral status and functional outcome	Suggested clinical relevance but not an independent surrogate marker
Liu et al. (30)	FVH topography and revascularization in MCA occlusion	FVH spatial distribution was associated with vascular involvement and revascularization-related changes	FVH topography may provide additional information beyond FVH presence or extent
Reyes et al. (38)	Whether FVHs provide perfusion information without CTP/MRP	FVHs provided qualitative information regarding perfusion abnormalities in patients without dedicated perfusion imaging	FVHs may complement but cannot replace quantitative perfusion assessment
Wang et al. (40), Hu et al. (41)	Overall association between FVHs and clinical outcomes	FVHs were associated with functional outcomes after reperfusion therapies	Associations exist, but heterogeneity limits interpretation as an independent prognostic biomarker
Tsang et al. (36)	Interpretation of FVHs after EVT	Persistent posttreatment FVHs may indicate residual distal hemodynamic impairment despite successful reperfusion	FVH interpretation is phase-dependent before and after reperfusion

## 4 FVHs-DWI mismatch and ischemic tissue status

Imaging assessment of acute ischemic stroke has progressively evolved from simple identification of vascular occlusion toward comprehensive evaluation of ischemic tissue status. The central goal is to identify tissue with the potential for recovery beyond the irreversible infarct core. Conventional perfusion imaging evaluates hypoperfused regions using parameters such as cerebral blood flow, cerebral blood volume, and time-based delay metrics, providing important guidance for reperfusion therapy in extended treatment windows (21, 22). However, perfusion parameters may be affected by post-processing algorithms, threshold selection, and technical variations among imaging platforms, and may not always be readily available in routine clinical settings (21, 22). As an imaging phenomenon derived from conventional MRI sequences, FVHs-DWI mismatch has attracted increasing attention because it reflects the spatial relationship between abnormal vascular signals and diffusion-restricted lesions.

FVHs-DWI mismatch generally refers to a situation in which the distribution of distal hyperintense vessels observed on FLAIR imaging extends beyond the region of DWI hyperintensity, indicating that areas with abnormal vascular signals have not yet developed corresponding extensive diffusion abnormalities (19, 31). As illustrated in Figure 3, FVHs and DWI lesions may demonstrate different spatial distributions. This phenomenon suggests that the distal vascular bed beyond the occlusion may retain a degree of residual blood flow, while some regions have not yet developed diffusion abnormalities corresponding to the extent of tissue injury (19, 20). Haussen et al. (19) reported that distal FVHs were significantly associated with perfusion–diffusion mismatch in patients with acute ischemic stroke, suggesting that FVHs may provide an imaging clue regarding differences in tissue status. Subsequent studies evaluating FVHs extending beyond the DWI lesion further explored their relationship with ischemic penumbra, but demonstrated that FVHs should not be considered a direct surrogate of tissue viability because they primarily reflect vascular flow alterations (32). However, the sample size of that study was limited, and the relationship between FVHs and perfusion abnormalities was primarily based on correlation analysis. Therefore, FVHs cannot currently replace perfusion imaging for the identification of ischemic penumbra.

**Figure 3:**
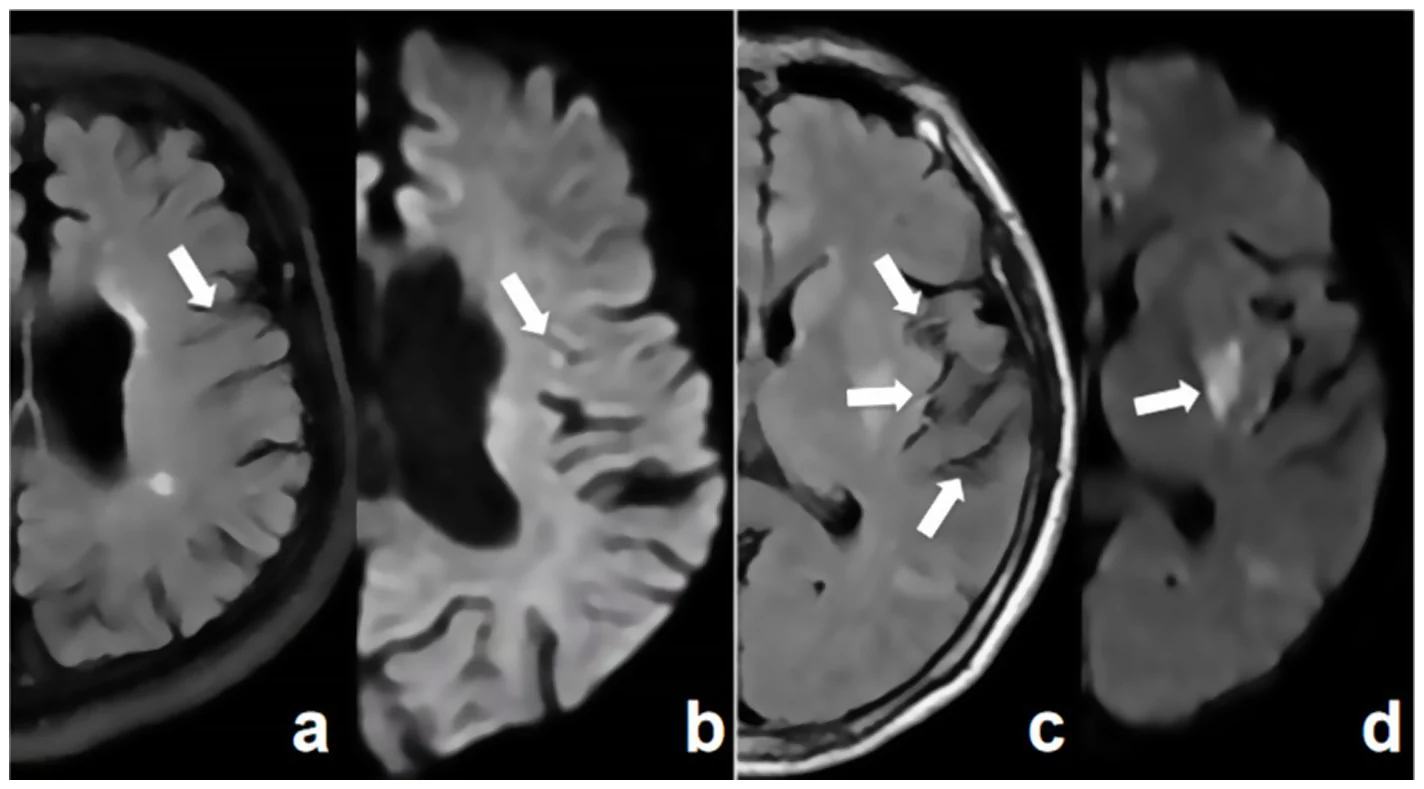
Qualitative assessment of FVHs-DWI mismatch. Panels **(a,b)**: FLAIR **(a)** and corresponding diffusion-weighted imaging (DWI) **(b)** demonstrate FVHs-DWI match, in which FVHs largely overlap with the regions of DWI hyperintensity. Panels **(c,d)**: FLAIR **(c)** and corresponding DWI **(d)** demonstrate FVHs-DWI mismatch, in which FVHs extend beyond the region of DWI hyperintensity. FVHs-DWI mismatch reflects a spatial discrepancy between abnormal vascular signals and diffusion abnormalities, indicating the coexistence of distal vascular flow alterations and diffusion abnormalities after acute arterial occlusion. This imaging pattern may suggest preserved but hemodynamically impaired distal circulation, although its relationship with tissue viability requires interpretation within a multimodal imaging framework.

The development of FVHs-DWI mismatch is closely related to the hemodynamic state after acute arterial occlusion (18–20, 31). After LVO, leptomeningeal collateral pathways allow partial blood flow to bypass the occluded segment and reach the distal vascular bed. However, because of longer collateral pathways and increased vascular resistance, blood reaching the distal circulation typically demonstrates reduced velocity and delayed arrival. Under these conditions, FLAIR imaging may reveal persistent abnormal hyperintense signals within distal vessels, whereas the extent of DWI abnormalities may not yet fully encompass the corresponding ischemic tissue. Therefore, FVHs-DWI mismatch may represent a state characterized by residual blood flow with hemodynamic limitation, rather than simply indicating either normally perfused tissue or irreversibly ischemic tissue (18, 20).

The relationship between FVHs-DWI mismatch and tissue recovery potential has received increasing attention in the era of reperfusion therapy. Legrand et al. (14) analyzed data from the THRACE trial and found that, among patients with proximal middle cerebral artery occlusion treated with mechanical thrombectomy, those with FVHs-DWI mismatch were more likely to achieve favorable functional outcomes after successful recanalization, suggesting an association between this imaging pattern and tissue recovery potential. However, this study represented a retrospective subgroup analysis of patients enrolled in a randomized treatment trial, with a limited sample size. Moreover, FVHs-DWI mismatch determination remains dependent on imaging interpretation criteria. Therefore, FVHs-DWI mismatch should currently be regarded as an adjunctive marker of tissue status rather than an independent criterion for treatment selection.

Recent studies have further expanded the potential implications of FVHs-DWI mismatch in tissue viability assessment and reperfusion decision-making. Boullet et al. (20) investigated patients with proximal middle cerebral artery occlusion and reported that FVHs-DWI mismatch was associated with the presence of ischemic penumbra, suggesting that this imaging pattern may capture the coexistence of residual distal blood flow and potentially salvageable tissue beyond the diffusion-defined infarct core. These findings support the hypothesis that FVHs-DWI mismatch may reflect a hemodynamic state characterized by preserved but impaired collateral-mediated perfusion rather than simply indicating tissue preservation.

More recently, Liu et al. (31) evaluated patients presenting beyond 24 h from last known well with FVHs-DWI mismatch and explored the association between endovascular thrombectomy and clinical outcomes in this selected population. Their findings suggested that FVHs-DWI mismatch may identify a subgroup with persistent hemodynamic support despite delayed presentation, highlighting its potential relevance in extended treatment windows. However, these observations require cautious interpretation because FVHs-DWI mismatch remains dependent on imaging assessment criteria, and prospective validation is needed before it can be incorporated into treatment selection algorithms.

FVHs-DWI mismatch should be distinguished from other mismatch concepts used in acute stroke imaging evaluation. DWI-FLAIR mismatch is primarily applied for estimating stroke onset time in patients with unknown symptom onset and is based on time-dependent differences between parenchymal DWI and FLAIR signal changes after ischemia; it does not reflect intravascular hemodynamic alterations (33). In contrast, FVHs-DWI mismatch focuses on the spatial relationship between abnormal vascular signals and tissue injury, reflecting the interaction between persistent vascular abnormalities and ischemic lesion extent. Large multicenter studies have demonstrated that FVHs themselves are associated with proximal arterial occlusion, larger initial ischemic lesions, and more severe neurological deficits, suggesting that FVH expression is influenced by both vascular status and stroke severity (18, 20, 34).

Compared with CT perfusion (CTP) or MR perfusion imaging, FVHs-DWI mismatch has advantages including no requirement for additional perfusion sequences and relatively simple acquisition. However, the information provided by FVHs remains limited (21, 22). CTP and MR perfusion mismatch analyses typically estimate the extent of hypoperfused tissue using quantitative cerebral blood flow parameters and delay thresholds, whereas FVHs primarily reflect abnormal intravascular flow conditions and cannot directly provide tissue-level perfusion measurements (21). Therefore, FVHs-DWI mismatch should not be considered a substitute for conventional perfusion-based penumbral assessment, but rather a complementary imaging feature providing information regarding hemodynamic alterations.

From the perspective of clinical decision-making in the reperfusion era, FVHs-DWI mismatch may provide additional information for identifying patients with residual circulatory support and potential tissue recovery capacity, particularly when perfusion imaging cannot be completed promptly or yields uncertain results. Nevertheless, its clinical application requires further validation through larger prospective studies, including standardized assessment criteria, direct comparison with CTP/MR perfusion parameters, and evaluation of predictive performance across different treatment windows (20).

Currently, FVHs-DWI mismatch is best understood as an imaging manifestation of the relationship between abnormal hemodynamics and tissue injury during acute ischemia. Its potential value does not lie in replacing established perfusion assessment methods, but rather in complementing information regarding residual flow, collateral contribution, and the interaction between cerebral circulation and tissue status. These emerging findings further support investigation of FVHs-DWI mismatch as a potential marker of collateral-mediated hemodynamic adaptation and tissue viability, while its incremental value for treatment selection remains to be determined (20).

## 5 Clinical applications of FVHs in the reperfusion era

With the emergence of mechanical thrombectomy as the standard treatment for acute LVO-related stroke, imaging evaluation has gradually evolved from simply confirming vascular occlusion toward comprehensive assessment of vascular status, tissue condition, and potential treatment benefit. Several randomized controlled trials have demonstrated that patients selected by imaging criteria indicating a small infarct core and substantial salvageable tissue can still achieve significant benefit from reperfusion therapy beyond conventional treatment windows (1–3). Currently, clinical decision-making primarily relies on CTA, CTP, MR perfusion, and diffusion-weighted imaging (DWI), which provide complementary information regarding vascular anatomy, tissue perfusion, and infarct core status. As an abnormal vascular signal pattern visible on conventional MRI sequences, FVHs may provide additional information related to distal hemodynamic conditions within the existing imaging framework.

The relationship between FVHs and functional outcomes after reperfusion therapy has received increasing attention. Earlier studies in patients receiving thrombolysis demonstrated associations between FVHs, hemodynamic abnormalities, and treatment response, suggesting that FVHs may capture clinically relevant distal flow alterations during acute ischemia (35). Nave et al. (13) analyzed patients with M1 segment middle cerebral artery occlusion who underwent endovascular treatment and found that FVH scores were associated with angiographic collateral status and 3-month functional outcomes, suggesting that FVHs may contain hemodynamic information related to tissue recovery after reperfusion. Further studies have demonstrated that FVH expression is influenced by multiple factors, including the location of vascular occlusion, infarct extent, and stroke severity. Using multicenter data, Cheng et al. (34) reported that FVHs were associated with proximal arterial occlusion, larger initial ischemic lesions, and more severe neurological deficits, indicating that FVHs should be interpreted in conjunction with clinical characteristics and other imaging findings. Therefore, future studies should not only further clarify the relationship between FVHs and clinical outcomes but also determine whether FVHs provide additional predictive value beyond existing imaging models.

FVHs-DWI mismatch has further expanded the potential clinical applications of FVHs in the reperfusion era. Legrand et al. (14) analyzed data from the THRACE trial and found that, among patients with proximal middle cerebral artery occlusion treated with mechanical thrombectomy, those with FVHs-DWI mismatch showed a trend toward better functional outcomes after successful recanalization, suggesting that this imaging pattern may be associated with greater tissue recovery potential (15). However, current evidence is mainly derived from exploratory analyses, with limited sample sizes and imaging interpretation-dependent assessment criteria. Therefore, FVHs-DWI mismatch is currently insufficient to serve as an independent criterion for treatment selection. Further multicenter studies are required to determine its predictive value across different treatment windows and occlusion subtypes.

An important consideration is that the biological meaning of FVHs may differ before and after reperfusion therapy. In the pre-reperfusion setting, FVHs primarily reflect the hemodynamic adaptation induced by arterial occlusion, including collateral recruitment, maintenance of residual flow, and delayed distal blood transit. Therefore, the proposed concept of a dynamic hemodynamic imaging phenotype mainly refers to this occlusion-related adaptive process. After successful reperfusion, however, the hemodynamic environment is substantially altered, and persistent or newly observed FVHs may reflect different mechanisms, such as incomplete restoration of distal flow, residual hemodynamic impairment, or post-reperfusion vascular changes. Recent studies have further suggested that posttreatment FVHs may refine the interpretation of angiographic reperfusion status by identifying residual distal hemodynamic abnormalities despite successful macrovascular reperfusion (36). Accordingly, FVHs after treatment should not be interpreted identically to pretreatment FVHs, and further studies are required to determine whether the same imaging phenotype framework applies across different treatment phases.

The potential clinical value of FVHs primarily lies in their complementary role within established multimodal imaging strategies. CTA and DSA mainly assess vascular structure, collateral pathways, and filling characteristics (4, 5), whereas multiphase CTA can additionally provide dynamic information regarding contrast arrival timing and collateral recruitment patterns (37). In contrast, FVHs may reflect the downstream hemodynamic consequences of collateral-mediated residual flow, including slow flow and delayed distal filling.

Whether FVHs provide incremental information beyond CTA and CTP remains an important unresolved question. CTA and CTP currently represent established components of acute stroke imaging workflows, providing information regarding vascular occlusion, collateral status, and quantitative tissue perfusion (5, 21, 22, 37). In contrast, FVHs do not directly measure tissue perfusion but may provide complementary information regarding distal hemodynamic consequences of collateral-mediated residual flow, including slow flow and delayed filling (6, 7, 12, 17, 18). Therefore, the potential value of FVHs may lie not in replacing CTA/CTP-based assessment, but in integrating additional information regarding distal vascular adaptation. However, whether FVH-related parameters improve predictive models beyond conventional imaging markers requires prospective validation (13, 14, 18, 34).

CTP and MR perfusion estimate the extent of hypoperfused tissue through quantitative perfusion parameters. In contrast, FVHs primarily reflect signal alterations associated with reduced distal intravascular flow and delayed filling, providing information related to abnormal hemodynamic states after arterial occlusion (6, 10, 15, 16). Therefore, FVHs should be interpreted as a complementary imaging feature within multimodal assessment rather than an alternative to established perfusion evaluation (21, 22).

The relationship between FVHs and multiphase CTA (mCTA) deserves particular consideration because CT-based imaging remains the primary modality for initial evaluation of acute stroke in many clinical settings. Both mCTA and FVHs provide information related to collateral-mediated hemodynamic alterations; however, they capture different aspects of this process. mCTA primarily evaluates the temporal characteristics of contrast arrival within collateral vessels, including collateral extent, filling delay, and dynamic collateral recruitment. In contrast, FVHs represent abnormal intravascular signal changes on FLAIR imaging associated with slow distal blood flow, prolonged transit time, and delayed filling after collateral recruitment. Therefore, FVHs should not be considered a substitute for mCTA-based collateral assessment. Rather, they may provide complementary information regarding the hemodynamic consequences of collateral circulation at the distal vascular level, particularly when residual flow is maintained but remains kinetically impaired.

However, whether FVHs provide clinically meaningful incremental information beyond mCTA remains uncertain. Future studies directly comparing FVHs with dynamic collateral parameters derived from mCTA are required to determine whether FVHs contribute additional value for tissue outcome assessment, treatment response prediction, or patient stratification.

Beyond collateral assessment, FVHs may provide complementary information regarding distal hemodynamic consequences of collateral recruitment, particularly when residual flow is maintained but remains kinetically impaired or when perfusion imaging is unavailable. These potential roles remain hypothesis-generating and require validation in prospective studies.

In extended-window reperfusion therapy, current imaging selection strategies are primarily based on mismatch concepts integrating infarct core size, clinical severity, and tissue perfusion status. The DAWN trial selected patients based on a clinical deficit–infarct core mismatch, whereas the DEFUSE 3 trial used perfusion imaging to identify patients with substantial ischemic penumbra (1, 2). Because FVHs can be assessed using conventional MRI sequences, they may serve as an additional source of information within multimodal imaging evaluation, particularly in situations where perfusion imaging cannot be obtained promptly or provides uncertain results. Studies have explored whether FVHs may provide qualitative information regarding perfusion abnormalities in patients without dedicated perfusion imaging (38). However, sufficient evidence is still lacking to demonstrate that FVHs independently improve the accuracy of treatment benefit prediction.

Future research on FVHs should focus on multimodal imaging integration and automated analysis. By combining FVHs assessment with CTA-based collateral evaluation, CTP/MR perfusion parameters, DWI-defined infarct core, and clinical variables, future studies may further clarify the role of FVHs in tissue status evaluation. Current limitations include the lack of standardized FVHs scoring systems, variability in MRI acquisition parameters, and insufficient prospective multicenter validation studies (13, 14, 34).

Therefore, in the era of reperfusion therapy, FVHs are better considered a complementary imaging phenotype reflecting distal abnormal flow conditions and collateral-mediated hemodynamic adaptation, which may provide complementary information regarding distal hemodynamic states beyond conventional assessment of collateral anatomy and tissue-level perfusion (12, 17, 31, 39). Recent meta-analyses have demonstrated associations between FVHs and functional outcomes after reperfusion therapies, including endovascular treatment and thrombolysis; however, the prognostic interpretation of FVHs remains limited by heterogeneity in patient populations, imaging timing, and FVH assessment methods (40, 41). However, the prognostic interpretation of FVHs remains limited by heterogeneity in patient populations, imaging timing, and FVH assessment methods (40). Their clinical utility requires further clarification through standardized evaluation methods and prospective clinical studies.

## 6 Future directions and challenges of FVHs research

Previous studies have demonstrated that FVHs are associated with vascular occlusion status, collateral circulation characteristics, tissue injury patterns, and clinical outcomes after reperfusion therapy in acute ischemic stroke. However, FVHs remain primarily investigated as an imaging finding rather than an established imaging biomarker with well-defined clinical applications (12–17, 34, 39). Future research should not only further investigate the clinical associations of FVHs but also clarify their biological significance, establish reproducible assessment systems, and determine their incremental value within current stroke imaging frameworks (12–14, 34, 39).

A major prerequisite for the further development of FVHs is the establishment of a standardized and reliable evaluation systems. Currently, FVHs are mainly defined by hyperintense vascular signals on FLAIR imaging, with assessment methods including FVH presence or absence, vessel counts, distribution extent, and regional scoring approaches. However, standardized criteria remain lacking across studies (6, 13, 25). Future development of FVHs requires progression from qualitative assessment toward standardized and reproducible quantitative evaluation (13).

Future development of FVHs requires progression from qualitative evaluation toward standardized and reproducible quantitative assessment. Current scoring approaches remain inconsistent regarding vessel counts, spatial distribution, and regional segmentation. Furthermore, MRI acquisition parameters, imaging protocols, and reader experience may influence FVH detection and quantification. Therefore, future studies should integrate automated analysis methods with multicenter validation to establish robust FVH evaluation systems with high reproducibility (Figure 4).

**Figure 4:**
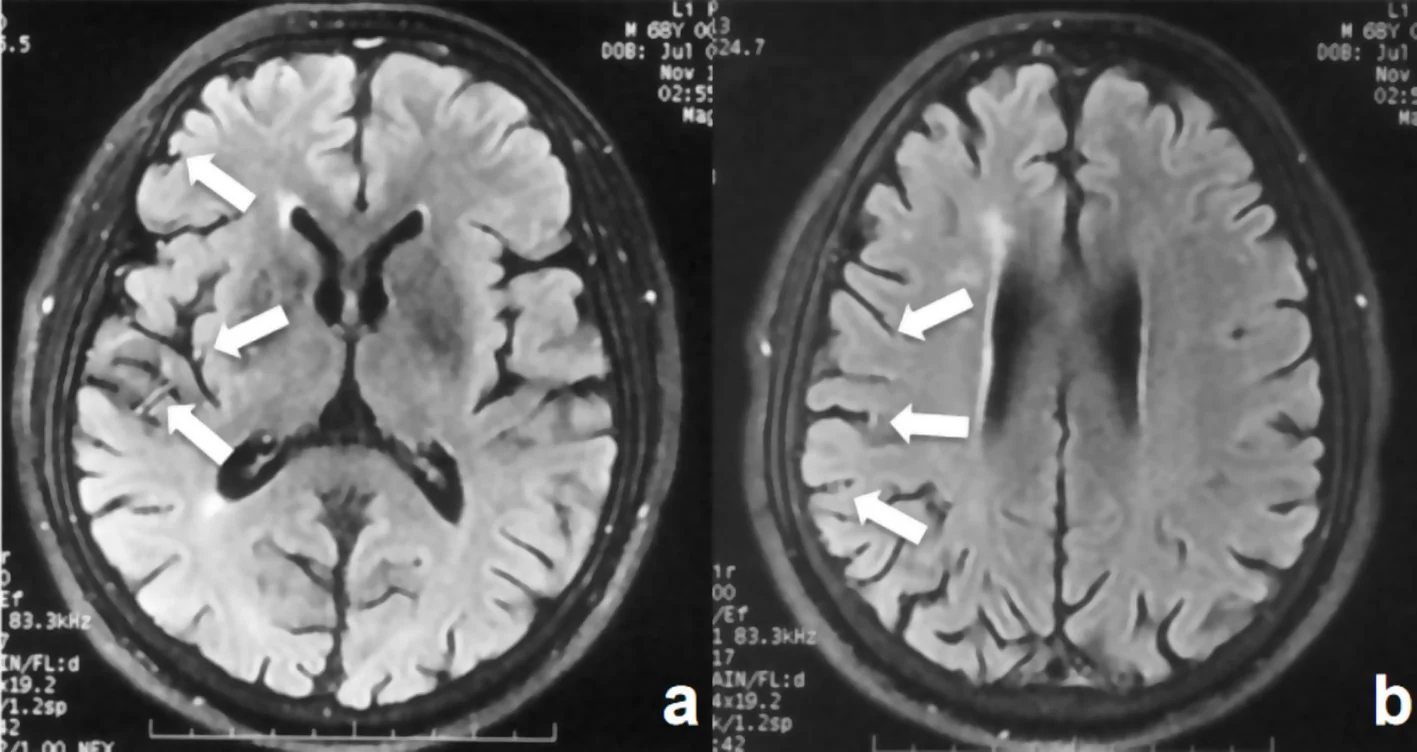
Regional assessment approach for FVH distribution. Panel **(a)**: Basal ganglia level demonstrating FVHs in the insular region (I), anterior MCA cortical territory (M1), and lateral insular cortex (M2). Panel **(b)**: Lateral ventricular level demonstrating FVHs in the cortex adjacent to the lateral insular cortex (M5) and posterior MCA territory (M6). A total of five regions demonstrate FVHs, corresponding to a semi-quantitative FVHs score of 5. This score reflects the extent of FVH distribution based on the presence or absence of FVHs in predefined anatomical regions and should not be interpreted as equivalent to conventional ASPECTS or as an independent indicator of favorable or unfavorable hemodynamic status.

A critical step toward clarifying the clinical significance of FVHs is to better understand the physiological processes represented by this imaging phenotype. Current evidence suggests that FVHs are associated with reduced distal blood flow velocity, delayed filling, and collateral-related hemodynamic states; however, FLAIR vascular hyperintensity itself does not directly reflect tissue perfusion or metabolic status (6, 21). Toyoda et al. (7) proposed that reduced blood flow velocity and prolonged blood residence time contribute to the formation of FLAIR vascular hyperintensity, whereas Lee et al. (12) further demonstrated an association between FVHs and collateral circulation characteristics in acute ischemic stroke. Future studies should combine CTP, perfusion MRI, arterial spin labeling, and dynamic blood flow parameters from DSA to further validate the relationship between FVHs, actual cerebral blood flow, and tissue perfusion reserve, thereby defining the specific physiological processes represented by FVHs.

Although FVHs have accumulated substantial evidence regarding clinical associations, whether they provide incremental information beyond existing imaging approaches remains uncertain. In patients undergoing reperfusion therapy, CTA, CTP, MR perfusion, and DWI already provide important information regarding vascular status, tissue perfusion, and infarct core characteristics. Therefore, future investigations should move beyond determining whether “FVHs are associated with outcomes” toward evaluating whether “FVHs provide independent incremental value” (15, 16). Prospective multicenter studies comparing prediction models with and without FVH-related parameters are needed to evaluate whether FVHs can improve patient stratification, prognostic assessment, or prediction of treatment response.

The pathophysiological process of acute ischemic stroke involves multiple interacting components, including vascular occlusion, collateral recruitment, blood flow redistribution, and evolution of tissue injury. Future imaging research should therefore integrate information across multiple dimensions of vascular status, hemodynamics, and tissue response. FVHs primarily provide information regarding abnormal distal hemodynamic conditions, whereas CTA, CTP/MR perfusion, and DWI reflect vascular anatomy, tissue perfusion, and infarct core status, respectively. Multimodal imaging integration may help further elucidate the dynamic interactions among vascular structure, blood flow, and tissue injury.

Accordingly, future studies should focus on evaluating the incremental contribution of FVHs within multimodal imaging models rather than applying FVHs as an isolated patient selection tool. Advances in artificial intelligence may provide new opportunities for automated FVH detection and quantitative assessment; however, such models require validation using large, multicenter datasets (42). Integration of multidimensional imaging information and optimization of standardized clinical workflows may further improve the efficiency of acute stroke assessment (43).

Overall, FVHs can currently be considered a candidate imaging phenotype reflecting dynamic hemodynamic adaptation. Their future development does not depend on replacing existing imaging techniques, but rather on defining their precise role within comprehensive acute ischemic stroke imaging through standardized evaluation, mechanistic validation, and assessment of incremental clinical value.

## 7 Conclusion

FVHs are common vascular-related signal abnormalities observed on MRI in patients with acute ischemic stroke. Their formation is closely associated with reduced distal blood flow velocity, prolonged blood transit time, and collateral-mediated alterations in distal hemodynamic conditions. Research on FVHs has gradually expanded from simple description of an imaging sign toward exploration of their associations with hemodynamic states, tissue injury, and outcomes after reperfusion therapy.

Current evidence suggests that FVHs may represent a candidate imaging phenotype reflecting physiological hemodynamic adaptation after acute arterial occlusion. Rather than an established clinical biomarker. Their clinical applicability requires further clarification through standardized assessment methods and prospective validation. Future research should address several key challenges, including the lack of unified evaluation criteria, incomplete understanding of their underlying biological mechanisms, and uncertainty regarding their incremental value within multimodal imaging frameworks.

The future role of FVHs does not lie in replacing established imaging techniques such as CTA, CTP, MR perfusion, or DWI, but rather in providing complementary information within comprehensive imaging evaluation for acute ischemic stroke. Through imaging standardization, validation of hemodynamic mechanisms, and prospective clinical studies, FVHs may further elucidate the dynamic interactions among vascular status, blood flow, and tissue response during acute ischemia, thereby providing new directions for precision stroke imaging.

## Literature search strategy

A literature search was performed in PubMed/MEDLINE, Embase, and Web of Science databases to identify relevant studies regarding FLAIR vascular hyperintensities (FVHs) in acute ischemic stroke. Search terms included combinations of “FLAIR vascular hyperintensities,” “hyperintense vessels,” “acute ischemic stroke,” “large vessel occlusion,” “collateral circulation,” and “hemodynamic.” Relevant articles published from database inception to March 2026 were considered, with emphasis on studies investigating FVH mechanisms, collateral relationships, tissue status, and clinical implications.
